# Double-orifice mitral valve associated with atrioventricular canal defects

**DOI:** 10.11604/pamj.2016.23.199.8676

**Published:** 2016-04-15

**Authors:** Jaafar Rhissassi, Hicham El Malki, Fatima Azzahra Benmessaoud, Tahar El Kandoussi, Mohamed Laaroussi

**Affiliations:** 1 Department of Cardiac Surgery A, Ibn Sina Hospital, Rabat, Morocco; 2 Department of Cardiac Surgery, Nouvel Hopital Civil, Strasbourg, France

**Keywords:** Mitral valve, double-orifice, atrioventricular canal defects

## Abstract

A 4 year-old male presented with effort dyspnea, and was diagnosed as atrioventricular canal defects. This finding was confirmed by open heart surgery, and a congenital double orifice mitral valve was discovered. The septal defect was closed but the double orifice mitral valve was respected because of the absence of hemodynamic disturbance. We report this case with review of literature.

## Introduction

Double orifice mitral valve (DOMV) is a rare congenital malformation characterized by a mitral valve with two orifices. This anomaly is usually detected with other cardiac malformation [1]. Although double orifice mitral valve may allow normal blood flow between the left atrium and LV, it can substantially obstruct mitral valve inflow or produce mitral valve incompetence [2]. Following is a case of DOMV; we have experienced with atrioventricular (AV) canal defects.

## Patient and observation

**Figure 1:**
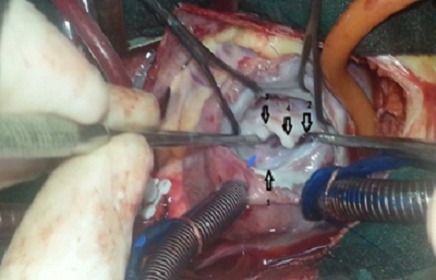
Intraoperative view of the mitral valve from the left atrial side shows: 1-2: different mitral orifices; 3: mitral cleft; 4: bridge

**Figure 2:**
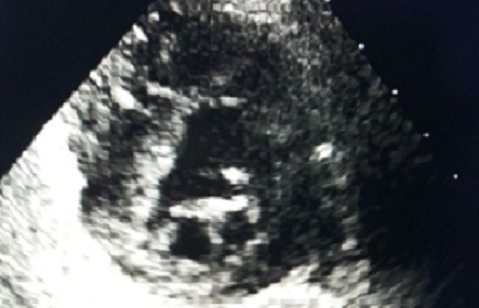
Postoperative echocardioqraphy

A four-year old male child, product of non-consanguineous marriage, with history of effort dyspnea and recurrent respiratory infection. On physical examination, a systolic murmur was heard in the left parasternal area. Echocardiography provided a diagnosis of atrioventricular (AV) canal defects. In the operating room, the primumatrial septal defect (ASD) and the mitral cleft were seen. A congenital DOMV was fortuitously discovered (Figure 1). The primum ASD was closed by a patch and the mitral cleft was partially closed in the summit. The DOMV was respected because of the absence of hemodynamic disturbance (Figure 2).

## Discussion

DOMV is a rare disorder that was first reported by Greenfield in 1876 [3]. This anomaly is usually detected with other cardiac malformation [1]. Rosenberg et al reported that 25% of patients with DOMV have partial persistent AV canal and about 5% of patients with partial persistent AV canal have DOMV [4]. In our case, there was a partial persistent AV canal as associated anomaly. In a DOMV, abnormal tissue divides the mitral orifice into two orifices. Baño-Rodrigo et al reported that the frequency of equalsized orifices in patients with DOMV is limited to 15% [5]. Echocardiographic classification was proposed by Trowitsch et al, which divided DOMV into 3 different types: a: hole type (accessory orifice surrounded by leaflet tissue that may have a chordal ring), b: complete bridging (fibrous bridge in the plane of the mitral valve sails, dividing the mitral valve opening into 2 parts that may be equal or unequal), and c: incomplete bridging (small strand of fibrous tissue connecting only the tips of the anterior and posterior leaflets) [6]. In our case, incomplete bridging was the type of DOVM malformations. Hemodynamic disturbance is not observed in most cases of DOMV, but in some patients’ significant mitral stenosis or regurgitation are associated [6]. Bibhuti et al reported that the mitral valve was functionally normal in 9 patients (50%) and only mildly impaired in 7(38%), and that only 2 patients (11%) had severe mitral regurgitation or stenosis [7]. In our case, a small mitral regurgitation is associated. Treatment is only necessary if significant mitral stenosis or mitral regurgitation is present or if repair of an associated cardiac lesion is needed. In our case the defective septal was closed in the operation.

## Conclusion

Transthoracic echocardiographic examination is a reliable, and in most cases sufficient, means of diagnosing DOMV and determining its type. This case demonstrates the necessity of careful imaging of the mitral valve apparatus in patients with AV canal defects.
